# Application of NOMA in Wireless System with Wireless Power Transfer Scheme: Outage and Ergodic Capacity Performance Analysis

**DOI:** 10.3390/s18103501

**Published:** 2018-10-17

**Authors:** Dinh-Thuan Do, Chi-Bao Le

**Affiliations:** 1 Wireless Communications Research Group, Faculty of Electrical and Electronics Engineering, Ton Duc Thang University, Ho Chi Minh City 700000, Vietnam; 2 Faculty of Electronics Technology, Industrial University of Ho Chi Minh City (IUH), Ho Chi Minh City 700000, Vietnam; baole.iuh@gmail.com

**Keywords:** non-orthogonal multiple access, energy harvesting, outage probability

## Abstract

Non-orthogonal multiple access (NOMA) and energy harvesting (EH) are combined to introduce a dual-hop wireless sensor system. In particular, this paper considers a novel EH protocol based on time power switching-based relaying (TPSR) architecture for amplify-and-forward (AF) mode. We introduce a novel system model presenting wireless network with impacts of energy harvesting fractions and derive analytical expressions for outage probability and ergodic rate for the information transmission link. It confirmed that the right selection of power allocation for NOMA users can be performed to obtain optimal outage and ergodic capacity performance. Theoretical results show that, in comparison with the conventional solutions, the proposed model can achieve acceptable outage performance for sufficiently small threshold signal to noise ratio (SNR) with condition of controlling time switching fractions and power splitting fractions appropriately in considered TPSR protocol. We also examine the impacts of transmitting power at source, transmission rate, the other key parameters of TPSR to outage, and ergodic performance. Simulation results are presented to corroborate the proposed system.

## 1. Introduction

In fifth generation (5G) wireless networks, one main expectation is to enhance energy efficiency significantly in comparison with previous generation networks. However, a large number of devices will be connected in future wireless networks where challenges with the explosion of mobile internet applications and Internet of things (IoT) services will be met. In addition, big concerns are highlighted as critical environmental issues, i.e., high carbon emissions. Therefore, such mass connections will unavoidably give rising global energy consumption with an unprecedented surge. Hence, green communication needs to be established to improve the network energy effectiveness. Inspired from advantages of various energy harvesting (EH) architectures, relaying network is proposed to provide information transmission and energy-transmission cooperation and it has now been suggested to improve the overall energy efficiency [1,2,3]. The popular energy resources in EH including solar and wind, which are intermittent under impacts the environmental alteration. Among the emerging technologies, radio-frequency (RF) signals have been regarded as viable new sources for EH. In [1,2], it was shown that RF EH can exhibit outage performance as introducing the energy efficiency of the wireless relaying networks. Furthermore, due to the dual properties of RF, namely information transmission and EH, an emerging technique termed as simultaneous wireless information and power transfer (SWIPT) has attracted increasing attention [3,4,5,6]. The authors in [3] first proposed power splitting and time switching schemes to wireless power transfer from the source to relay to guarantee operation of the second hop in dual-hop relaying network. More importantly, outage probability and ergodic capacity are popular metrics to evaluate system performance. The authors in [4] investigated SWIPT over deploying new energy harvesting originated from co-channel interference. Similar trend to explore SWIPT more is that tractable form of derived expressions for performance analysis [5] and then extended work can be reported in [6] into a practical imperfect channel state information (CSI) to consider optimal policy regrading instantaneous rate. The impact of energy harvesting on performance of relaying policies regarding scenario of fixed power allocation and cognitive radio is considered and such a model is deployed with simultaneous wireless information and power transfer [7].

Recently, non-orthogonal multiple access (NOMA) has been suggested to adapt the explosive progress of mobile Internet regarding data traffic volume [8,9,10]. To realize higher spectral effectiveness than conventional orthogonal multiple access, i.e., orthogonal frequency division multiple access (OFDMA), both successive interference cancelation (SIC) and superposition coding scheme are applied respectively at the transmitters and the receivers in NOMA. Besides, in the same frequency or time resources, NOMA’s users can more connections can be supported by allowing simultaneously access [11,12,13,14]. Consequently, the next generation of mobile communication systems can deploy NOMA as a favorable multiple access scheme for [15,16,17]. In addition, by splitting them in the power domain and in the same frequency band and time slot, multiple users in NOMA can be simultaneously served [18]. The authors in [19] presented basic concept of NOMA together with SIC receiver scheme. Considering the case of fixed power allocation, the authors in [20] investigated ergodic sum rate and the outage performance for NOMA.

In another line of research, the existing advanced schemes such as multiple-input multiple-output (MIMO) can be included in NOMA as an attractive property in research about NOMA [21]. In a different system model, NOMA is studied in cooperative relaying networks (CRS) [22], heterogeneous system [23], and device-to-device (D2D) networks with full-duplex scheme [24]. In another trend, the cooperative NOMA (C-NOMA) together with CRS as investigation in [24] in which spatially multiplexed scheme to serve a single user.

Motivated by these discussions, in this paper we propose and analyse a relaying wireless sensor system with WPT under collecting energy from an external energy resource. Our results show that the TPSR factor and the transmission power at the relay should be jointly designed to achieve an optimal ergodic throughput efficiency, and system performance in NOMA outperforms than orthogonal multiple access (OMA) as investigations in the literature [20]. The main contributions of this paper can be shown as:

- Different with system model and mathematical analysis reported in [7], the new architecture related to EH (namely EH-NOMA protocol) is investigated and the impact of energy-aware fractions on wireless sensor system performance are studied. Such system model is designed as a combination of the two traditional EH receivers time switching based relay (TSR) and power splitting based relay (PSR) [3] in unique protocol, namely TPSR [25]. Although, we analysed system performance for OMA based network in [25] but this paper is extended work to highlight performance in NOMA scenario.
- We derive some analytical expressions of cumulative distribution function (CDF) of signal-to-noise ratio (SNR) and then outage probability is derived for system evaluation with considerations of power allocation factors in EH-NOMA protocol applied at the relay.
- The ergodic rate is derived to evaluate ergodic performance of such EH-NOMA protocol where energy harvesting-aware fractions are chosen reasonably to achieve better performance.
- To highlight advantages of NOMA, the traditional OMA and non-energy harvesting situation are presented. Such comparison can be observed in simulation results to confirm our analytical expressions.
- Next, Monte Carlo simulations are presented the outage performance to corroborate our analysis and the impact of some significant parameters on proposed protocol in EH-assisted networks are investigated.

The remainder of this paper is organized as follows: Section 2 presents the system model and TPSR protocol deployed in NOMA assisted wireless sensor system is investigated. In Section 3, we derive the analytical expressions of outage probability and ergodic throughput in delay-tolerant transmission. Section 4 examines the simulation results. Finally, Section 5 completes with conclusion remarks for the paper and reviews the important results.

**Notation:** Throughout this paper, $P_{r}\left(.\right)$ denotes probability, $F_{Z}\left(.\right)$ and $f_{Z}\left(.\right)$ symbolize the cumulative distribution function (CDF) and the probability density function (PDF) of a random variable *Z*, respectively, and $E\left\{.\right\}$ indicates the expectation operator.

## 2. System Model

**Figure 1:**
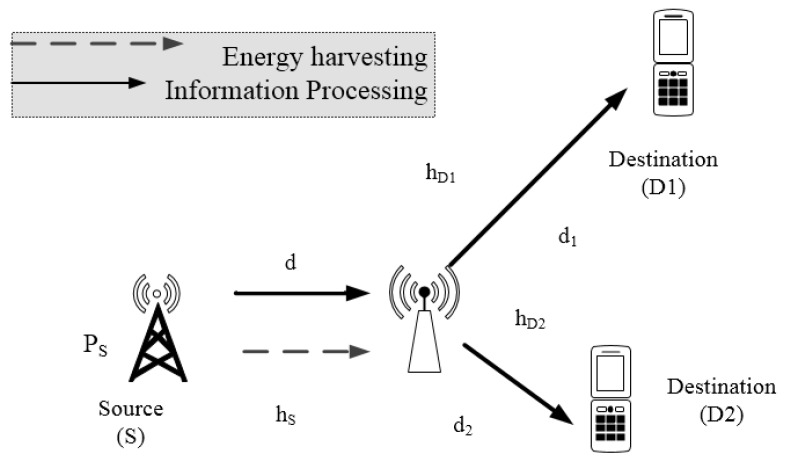
System model of energy harvesting (EH)-non-orthogonal multiple access (NOMA).

We consider cooperative AF relaying wireless sensor network, where the source $\left(S\right)$ communicates with two destinations $\left(D1,D2\right)$ through an intermediate relay $\left(R\right)$. Figure 1 shows relay scheme to source node can be served two far devices or sensor by requiring help of wireless powered relay. It is worth pointing out that these considered models can be deployed in wireless sensor or mobile network. The link between the source and the destination is unreliable or unavailable, so the transmission can only happen successfully with the aid of the wirelessly powered relay. In particular, the relay node deployed in this paper is characterized as energy-constrained node. Furthermore, each node is furnished with a single antenna, and half-duplex mode using amplify-and-forward (AF) strategy is deployed in the relay. We call $d,d_{1},d_{2}$ are distances between source and relay and relay to *D*1, *D*2 respectively; *m* is path loss coefficients. The relay acquires two independent data symbols during two time epochs, $x_{1}$ and $x_{2}$ transmitted from *S* directly and such signal processing need assistance of the relay, whereas the EH-NOMA relay delivers data symbol with harvested power.

Ernergy harvesting protocol for such NOMA scheme is time power based relaying energy harvesting (TPSR) protocol as recent investigation in [25]. In particular, during the first phase *S* broadcasts a superposition-coded signal $x_{s}$ (i.e., it equals to $a_{1}x_{1}+a_{2}x_{2}$) to the relay (i.e., *R*) and the destination $\left(D1,D2\right)$, where $P_{S}$ represents the total transmit power of the source *S*, and $a_{1},a_{2}$ symbolizes the power allocation quantity for symbol $x_{1},x_{2}$ respectively. The superimposed signal will be detached at destination for separated services in NOMA users, and without of generality it can be assumed $a_{1}>a_{2}$ to satisfy condition $a_{1}^{2}+a_{2}^{2}=1$. Due to similar performance at $\left(D1,D2\right)$, we assume that the following consideration aims to evaluate at (*D*1). We denote $h_{D}$ as channel between relay and (*D*1). The channel gains between the nodes are modeled as $h_{S}∼\;CN\left(0,\Omega_{1}\right)$ and two channels to *D*1 and *D*2 are assumed as the same average gains $h_{D1},h_{D2}∼CN\left(0,\Omega_{2}\right)$ due to *D*1, *D*2 belong to one group of NOMA users, and assuming that all channels follow flat fading Rayleigh distribution.

During the first phase, the received signals at the relay is given by:

(1) $$y_{R}=\frac{1}{\sqrt{d^{m}}}\sqrt{\left(1-\beta \right)P_{S}}h_{S}\left(a_{1}x_{1}+a_{2}x_{2}\right)+\sqrt{\left(1-\beta \right)}n_{R}^{A}+n_{R}^{C}$$

where $n_{R}^{A}∼CN\left(0,\sigma_{nR_{A}}^{2}\right)$ stands for antenna noise, $n_{R}^{C}∼CN\left(0,\sigma_{nR_{C}}^{2}\right)$ is complex additive white Gaussian noise (AWGN) at relay due to signal RF converting to base band signal. Using Amplify-and-Forward (AF) scheme, the relay will be amplified with factor *G* as given by:

(2) $$G=\frac{1}{\sqrt{\left(1-\beta \right)P_{S}{\left|h_{S}\right|}^{2}d^{-m}+\left(1-\beta \right)\sigma_{nA}^{2}+\sigma_{nC}^{2}}}$$

After signal processing at relay, in the second phase the relay amplifies the received signal to forward to destination with power as $P_{R}$, this power depends on harvested power in first phase. In such model, the role of *D*1 and *D*2 is similar, we only consider on performance of *D*1. The received signal at destination *D*1 denoted as $y_{D1}$ given by:

(3) $$y_{D1}=\frac{\sqrt{P_{R}}h_{D}G}{\sqrt{d_{1}^{m}}}y_{R}+n_{D}^{A}+n_{D}^{C}$$

where $n_{D}^{A}∼CN\left(0,\sigma_{nD_{A}}^{2}\right)$ and $n_{D}^{C}∼CN\left(0,\sigma_{nD_{C}}^{2}\right)$ are respectively white Gaussian noise (AWGN) at destination and converting operation to base band from RF signal at destination *D*1. Plugging $y_{R}$ and *G* from (1) and (2) into (3), $y_{D}$ can be expressed as:

(4) $$\begin{array}{cc} y_{D1} & =\frac{\sqrt{\left(1-\beta \right)P_{R}P_{S}}h_{S}h_{D1}\left(a_{1}x_{1}+a_{2}x_{2}\right)}{\sqrt{\left(1-\beta \right)P_{S}{\left|h_{S}\right|}^{2}d_{1}^{m}+d^{m}d_{1}^{m}\sigma_{nR}^{2}}}\\ & +\frac{\sqrt{P_{R}d^{m}}h_{D1}n_{R}}{\sqrt{\left(1-\beta \right)P_{S}{\left|h_{S}\right|}^{2}d_{1}^{m}+d^{m}d_{1}^{m}\sigma_{nR}^{2}}}+n_{D} \end{array}$$

We denote $n_{R}\overset{\Delta }{\;=}\sqrt{\left(1-\beta \right)}n_{R}^{A}+n_{R}^{C}$ and $n_{D}\overset{\Delta }{\;=}n_{D}^{A}+n_{D}^{C}$ as overall noise AWGN at the relay and destination. Therefore, $\sigma_{nR}^{2}\overset{\Delta }{\;=}\left(1-\beta \right)\sigma_{nR_{A}}^{2}+\sigma_{nR_{C}}^{2}$ is total variance noise AWGN at relay in TPSR. The harvested energy for signal processing at relay in energy harvesting phase denoted as $E_{h}^{TPSR}$ re-used in next signal processing $\left(1-\alpha \right)T,$ and hence the transmit power at relay can be computed as:

(5) $$P_{R}=\frac{E_{h}^{TPSR}}{\left(1-\alpha \right)T}=\eta \left(\frac{P_{S}{\left|h_{S}\right|}^{2}}{d^{m}}\right)\frac{\alpha \beta }{\left(1-\alpha \right)}$$

By replacing $P_{R}$ from (5) into (4), the received signal at destination *D*1 $y_{D1}$ can be given by:

(6) $$\begin{array}{cc} y_{D1} & =\underset{\mathrm{Signal}}{\underset{︸}{\frac{\sqrt{\eta \left(P_{S}{\left|h_{S}\right|}^{2}d_{1}^{m}\right)\alpha \beta \left(1-\beta \right)P_{S}}h_{S}h_{D1}x_{S}}{\sqrt{d^{m}d_{1}^{m}\left(1-\alpha \right)}\sqrt{\left(1-\beta \right)P_{S}{\left|h_{S}\right|}^{2}d_{1}^{m}+d^{m}\sigma_{nR}^{2}}}}}\\ & \underset{\mathrm{Noise}}{\underset{︸}{+\frac{\sqrt{\eta \left(P_{S}{\left|h_{S}\right|}^{2}d_{1}^{m}\right)\alpha \beta d^{m}}h_{D1}n_{R}}{\sqrt{d_{1}^{m}\left(1-\alpha \right)}\sqrt{\left(1-\beta \right)P_{S}{\left|h_{S}\right|}^{2}d_{1}^{m}+d^{m}\sigma_{nR}^{2}}}+n_{D1}}} \end{array}$$

## 3. Outage Performance and Ergodic Capacity Analysis

We first determine the signal to noise ratio (SNR) as the following section, and then we perform outage probability and then ergodic capacity is derived. Such outage event is evaluated as probability to the following system rate less than the pre-defined threshold rate Ith, i.e., $\mathrm{Pr}\left(I_{RD}\leq I\mathrm{th}\right)$:

(7) $$I_{RD}=\frac{1}{2}\log_{2}\left(1+SNR_{D}\right)$$

In particular, the end-to-end SNR can be computed by:

(8) $$SNR_{D}=\frac{E\{|Signal{|}^{2}\}}{E\{|Noise{|}^{2}\}}$$

where $E\left\{.\right\}$ stands for expectation operation. In this study, we denote AWGN noise terms at *D*1, *D*2 are the same, and equal to $\sigma_{nD}^{2}$. In principle of NOMA, the successive interference cancellation (SIC) will be implemented at each user in dedicated group to decode at each user and separate superimposed symbols and traditional OMA can mitigate the inter-user interference as well. At destination *D*1, the considered user first consider *D*2’s signal as noise to detect its own signal, while SIC is deployed at *D*2 to help detection of *D*2’ signal. In order to realize a stable trade-off between system throughput and user fairness, it is known that assigning less transmit power for users with better channel conditions and greater transmit power for users with worse channel conditions. In NOMA, the coding order is determined based on QoS requirement.

Without loss of generality, we further assume that following the principle of NOMA, the users’ power allocation coefficients are ordered as $a_{1}^{2}>a_{2}^{2}$ [26]. In general, in order to ensure the performance of NOMA systems, the NOMA-strong user (i.e., *D*1) is allocated less power than the NOMA weak user (i.e., *D*2). As a result, it can be obtained specific SNR for detect *D*1’ signal at *D*1 as:

(9) $$SNR_{D1,x1}=\frac{\eta \alpha \beta \left(1-\beta \right)P_{S}{\left|h_{S}\right|}^{2}{\left|h_{D1}\right|}^{2}a_{1}^{2}}{\eta \alpha \beta \left(1-\beta \right)P_{S}{\left|h_{S}\right|}^{2}{\left|h_{D1}\right|}^{2}a_{2}^{2}+\eta \alpha \beta d_{}^{m}{\left|h_{D1}\right|}^{2}\sigma_{nR}^{2}+Q_{1}+\frac{\left(1-\alpha \right)d^{m}d_{1}^{m}\sigma_{nR}^{2}\sigma_{nD}^{2}}{P_{S}{\left|h_{S}\right|}^{2}}}$$

where $Q_{1}=\left(1-\alpha \right)\left(1-\beta \right)d^{m}d_{1}^{m}\sigma_{nD}^{2}$

The simple form of (9) needs to be derived for the next calculation. The reason for simplify above expression is that small component can be eliminated, i.e., $\frac{\left(1-\alpha \right)d^{m}d_{1}^{m}\sigma_{nR}^{2}\sigma_{nD}^{2}}{P_{S}{\left|h_{S}\right|}^{2}}$ at high SNR regime. As a result, at high SNR, the received SNR for detecting symbols $x_{1}$ at the destination D1 in approximate value can be given as:

(10) $$SNR_{D1,x1}\approx \frac{\eta \alpha \beta \left(1-\beta \right)P_{S}{\left|h_{S}\right|}^{2}{\left|h_{D1}\right|}^{2}a_{1}^{2}}{\eta \alpha \beta d^{m}{\left|h_{D1}\right|}^{2}\sigma_{nR}^{2}+\eta \alpha \beta \left(1-\beta \right)P_{S}{\left|h_{S}\right|}^{2}{\left|h_{D1}\right|}^{2}a_{2}^{2}+Q_{1}}$$

Different with decoding operation in *D*1, it is worth noting that at *D*2 noise firstly needs to be eliminated. In this case, the signal of *D*1 is considered as noise. As a result, the received SNR for detect noise term $x_{1}$ at *D*2 given by:

(11) $$SNR_{D2,x1}\approx \frac{\eta \alpha \beta \left(1-\beta \right)P_{S}{\left|h_{S}\right|}^{2}{\left|h_{D2}\right|}^{2}a_{1}^{2}}{\eta \alpha \beta \left(1-\beta \right)P_{S}{\left|h_{S}\right|}^{2}{\left|h_{D2}\right|}^{2}a_{2}^{2}+\eta \alpha \beta d_{2}^{m}{\left|h_{D2}\right|}^{2}\sigma_{nR}^{2}+Q_{2}}$$

where $Q_{2}=\left(1-\alpha \right)\left(1-\beta \right)d^{m}d_{2}^{m}\sigma_{nD}^{2}$.

Following principle of NOMA, after SIC operation occurs at destination *D*2, the receiving SNR for detecting $x_{2}$ is given by:

(12) $$SNR_{D2,x2}=\frac{\eta \alpha \beta \left(1-\beta \right)P_{S}{\left|h_{S}\right|}^{2}{\left|h_{D2}\right|}^{2}a_{2}^{2}}{\eta \alpha \beta d_{2}^{m}{\left|h_{D2}\right|}^{2}\sigma_{nR}^{2}+\left(1-\alpha \right)\left(1-\beta \right)d^{m}d_{2}^{m}\sigma_{nD}^{2}}$$

### 3.1. Outage Probability

The following consideration provides an exact expression for the outage probability achieved by the two-stage AF relay in the proposed EH-NOMA. More importantly, the outage probability for such EH-NOMA with the proposed TPSR relaying scheme for *D*1, *D*2 are respectively calculated. For *D*1’ signal, an outage event for $x_{1}$ can be interpreted by main reason, i.e., it cannot detect its own message. For *D*2’ signal, the outage would happen for $x_{2}$ in two cases where *D*2 can not detect *D*1’s information and also can not recover its own information [26]. It is noted that in this paper direct link is not considered, then outage expressions are simpler than that in [26]. Such outage events for *D*1 and *D*2 can be given respectively by:

(13) $$\begin{array}{c} OP_{D1}=\underset{J2}{\underset{︸}{\mathrm{Pr}\left(SNR_{D1,x1}<\gamma_{1}\right)}}\\ and\\ OP_{D2}=\underset{J1}{\underset{︸}{\mathrm{Pr}\left(SNR_{D2,x1}<\gamma_{1}\right)}}\underset{J3}{\underset{︸}{\mathrm{Pr}\left(SNR_{D2,x2}<\gamma_{2}\right)}} \end{array}$$

In special case at high SNR, it can be shown the closed-form expression of remaining outage probabilities can be computed by applying following Lemma 1.

**Lemma** **1.** *We denote $\omega =\eta \alpha \beta d_{X}^{m}\sigma_{nR}^{2}\gamma ,$$\theta =\left(1-\alpha \right)\left(1-\beta \right)d_{X}^{m}d_{Y}^{m}\sigma_{nD}^{2}\gamma$ and $\psi =\eta \alpha \beta \left(1-\beta \right)P_{S}.$ The outage expression corresponding the threshold SNR γ can be expressed by:*

(14) $$\begin{array}{cc} OP_{out}^{TPSR} & =\mathrm{Pr}\left(\frac{\eta \alpha \beta \left(1-\beta \right)P_{S}{\left|h_{S}\right|}^{2}{\left|h_{D}\right|}^{2}}{\eta \alpha \beta {\left|h_{D}\right|}^{2}d_{X}^{m}\sigma_{nR}^{2}+\left(1-\alpha \right)\left(1-\beta \right)d_{X}^{m}d_{Y}^{m}\sigma_{nD}^{2}}<\gamma \right)\\ & =\mathrm{Pr}\left({\left|h_{D}\right|}^{2}<\frac{\theta }{\psi {\left|h_{S}\right|}^{2}-\omega }\right) \end{array}$$

*As a result, it can be solved in closed-form by:*

(15) $$\begin{array}{cc} \mathrm{Pr}\left(SNR<\gamma \right) & =1-\frac{1}{\psi \Omega_{1}}\exp\left(-\frac{\omega }{\psi \Omega_{1}}\right)\int_{\mu =0}^{\infty }\exp\left(-\frac{\mu }{\psi \Omega_{1}}-\frac{\theta }{\mu \Omega_{2}}\right)d\mu \\ & =1-\exp\left(-\frac{\omega }{\psi \Omega_{1}}\right)\sqrt{\frac{4\theta }{\psi \Omega_{1}\Omega_{2}}}K_{1}\left(\sqrt{\frac{4\theta }{\psi \Omega_{1}\Omega_{2}}}\right) \end{array}$$

*where $K_{1}\left(.\right)$ is first order Bessel function.*

**Proof.**  See in Appendix A. □

**Proposition** **1.** *The outage probability for D1 in EH-NOMA system given by:*

(16) $$OP_{D1}=1-\exp\left(-\frac{\omega_{1}}{\psi_{1}\Omega_{1}}\right)\sqrt{\frac{4\theta_{1}}{\psi_{1}\Omega_{1}\Omega_{2}}}K_{1}\left(\sqrt{\frac{4\theta_{1}}{\psi_{1}\Omega_{1}\Omega_{2}}}\right)$$

*where $\psi_{1}=\eta \alpha \beta \left(1-\beta \right)P_{S}\left(a_{1}^{2}-\gamma_{1}a_{2}^{2}\right)$, $\omega_{1}=\eta \alpha \beta d^{m}\sigma_{nR}^{2}\gamma_{1},$ and $\theta_{1}=\left(1-\alpha \right)\left(1-\beta \right)d^{m}d_{1}^{m}\sigma_{nD}^{2}\gamma_{1}$ are defined similarly in Lemma 1.*

**Proof** **of** **Proposition** **1.** Applying Lemma 1, it can be obtained outage event $OP_{D1}$ in (13) as below:

(17) $$\begin{array}{cc} J2 & =1-\frac{1}{\psi_{1}\Omega_{1}}\exp\left(-\frac{\omega_{1}}{\psi_{1}\Omega_{1}}\right)\int_{\mu =0}^{\infty }\exp\left(-\frac{\mu }{\psi_{1}\Omega_{1}}-\frac{\theta_{1}}{\mu \Omega_{2}}\right)d\mu \\ & =1-\exp\left(-\frac{\omega_{1}}{\psi_{1}\Omega_{1}}\right)\sqrt{\frac{4\theta_{1}}{\psi_{1}\Omega_{1}\Omega_{2}}}K_{1}\left(\sqrt{\frac{4\theta_{1}}{\psi_{1}\Omega_{1}\Omega_{2}}}\right) \end{array}$$

with $\psi_{1}$, $\omega_{1}$, and $\theta_{1}$ are defined below Proposition 1. This is the end of the proof. □

**Proposition** **2.** *The outage probability for D2 in EH-NOMA system given by:*

(18) $$\begin{array}{cc} OP_{D2}= & \left[1-\exp\left(-\frac{\omega_{1}}{\psi_{2}\Omega_{1}}\right)\sqrt{\frac{4\theta_{2}}{\psi_{2}\Omega_{1}\Omega_{2}}}K_{1}\left(\sqrt{\frac{4\theta_{2}}{\psi_{2}\Omega_{1}\Omega_{2}}}\right)\right]\\ & \times \left[1-\exp\left(-\frac{\omega_{1}}{\psi_{3}\Omega_{1}}\right)\sqrt{\frac{4\theta_{2}}{\psi_{3}\Omega_{1}\Omega_{2}}}K_{1}\left(\sqrt{\frac{4\theta_{2}}{\psi_{3}\Omega_{2}}}\right)\right], \end{array}$$

*where $\psi_{2}=\eta \alpha \beta \left(1-\beta \right)P_{S}\left(a_{1}^{2}-\gamma_{2}a_{2}^{2}\right)$, $\psi_{3}=\eta \alpha \beta \left(1-\beta \right)P_{S}a_{2}^{2}$, $\omega_{1}$, and $\theta_{2}=\left(1-\alpha \right)\left(1-\beta \right)d^{m}d_{2}^{m}\sigma_{nD}^{2}\gamma_{2}$ are defined similarly below Lemma 1.*

**Proof** **Proposition** **2.** Applying result from Lemma 1 and related formula, it is easy to obtain the final expression of $OP_{D2}$ in (13) J1 and J3 can be obtained by:

(19) $$\begin{array}{cc} J1 & =1-\frac{1}{\psi_{2}\Omega_{1}}\exp\left(-\frac{\omega_{1}}{\psi_{2}\Omega_{1}}\right)\int_{\mu =0}^{\infty }\exp\left(-\frac{\mu }{\psi_{2}\Omega_{1}}-\frac{\theta }{\mu \Omega_{2}}\right)d\mu \\ & =1-\exp\left(-\frac{\omega_{1}}{\psi_{2}\Omega_{1}}\right)\sqrt{\frac{4\theta }{\psi_{2}\Omega_{1}\Omega_{2}}}K_{1}\left(\sqrt{\frac{4\theta_{2}}{\psi_{2}\Omega_{1}\Omega_{2}}}\right) \end{array}$$

In similar way, the separated outage probability, J3, can be computed by:

(20) $$\begin{array}{cc} J3 & =1-\frac{1}{\psi_{3}\Omega_{1}}\exp\left(-\frac{\omega_{1}}{\psi_{3}\Omega_{1}}\right)\int_{\mu =0}^{\infty }\exp\left(-\frac{\mu }{\psi_{3}\Omega_{1}}-\frac{\theta_{2}}{\mu \Omega_{2}}\right)d\mu \\ & =1-\exp\left(-\frac{\omega_{1}}{\psi_{3}\Omega_{1}}\right)\sqrt{\frac{4\theta_{2}}{\psi_{3}\Omega_{1}\Omega_{2}}}K_{1}\left(\sqrt{\frac{4\theta_{2}}{\psi_{3}v\Omega_{2}}}\right), \end{array}$$

where $\omega_{1}$, $\theta_{2}$, $\psi_{2}$ and $\psi_{3}$ are defined below proposition 2. Replacing J1 and J3 into (13), Proposition 2 is determined. This ends the proof. □

**Remark** **1.** *It hard to derive optimal time switching and power splitting factor to achieve best outage performance as in (16) and (18). In fact, several algorithms are proposed to determine optimal throughput. However, time consuming for such computation exhibits to delay and hence system performance will be worse than the usual case. As a result, this paper only considers a couple of energy harvesting parameters in simulation to evaluate where the optimal scenario can be obtained.*

### 3.2. Ergodic Capacity

The achievable ergodic capacity of the proposed EH-NOMA system is examined, and we have the following key result. In this section, the ergodic achievable rate of $x_{1}$ at *D*1 can be readily calculated as:

(21) $$C_{D1}=E\left\{\log_{2}\left(1+SNR_{D1,x1}\right)\right\}$$

It is can be rewritten as below [3]:

(22) $$\begin{array}{c} C_{D1}=\int_{\gamma =0}^{\infty }\int_{z=d/c_{1}}^{\infty }\frac{\left(az\right)c_{1}z^{2}}{{\left(c_{1}z^{2}-dz\right)}^{2}\Omega_{1}\Omega_{2}y}\;e^{-\left(\frac{z}{\Omega_{1}}+\frac{az}{\left(c_{1}z^{2}-dz\right)\Omega_{2}}\right)}\log_{2}\left(1+\gamma \right)dzd\gamma \end{array}$$

in which $d=\eta \alpha \beta d^{m}\sigma_{nR}^{2}\gamma ,$ $a=\left(1-\alpha \right)\left(1-\beta \right)d^{m}d_{1}^{m}\sigma_{nD}^{2}\gamma$, $c_{1}=\eta \alpha \beta \left(1-\beta \right)P_{S}a_{2}^{2}.$

**Proposition** **3.** *The ergodic capacity at the D1 for the TPSR protocol is given by:*

(23) $$\begin{array}{c} C_{D1}\;\approx \int_{\gamma =0}^{\infty }\left(\frac{\varphi^{2}K_{0}\left(\varphi \right)e^{-\frac{d}{c_{1}\Omega_{1}}}}{2\gamma }+\frac{d\varphi K_{1}\left(\varphi \right)e^{-\frac{d}{c_{1}\Omega_{1}}}}{\gamma c_{1}\Omega_{1}}\right)\log_{2}\left(1+\gamma \right)d\gamma \end{array}$$

*where $\varphi^{2}=\frac{4a}{c\Omega_{1}\Omega_{2}}$*

*Similarly, the ergodic achievable rate of $x_{2}$ for D2 can be considered as:*

(24) $$C_{D2}=E\left\{\log_{2}\left(1+\min\left(SNR_{D2,x1},SNR_{D2,x2}\right)\right)\right\}$$

*To the best of the authors’ knowledge, the ergodic rate for D2 does not admit a closed-form expression. As a result, in case of high SNR, it can be obtained the closed-form as below.*

**Proposition** **4.** *The ergodic capacity at the D2 for the TPSR protocol is calculated by:*

(25) $$C_{D2}=\frac{1}{2}E\left\{1+\min\left(\frac{a_{1}^{2}}{a_{2}^{2}},\frac{a_{2}^{2}P_{S}{\left|h_{S}\right|}^{2}\left(1-\beta \right)}{d_{2}^{m}\sigma^{2}}\right)\right\}$$

*and it is shown in closed-form as:*

(26) $$\begin{array}{c} C_{D2}=\left[Ei\left(\frac{-d_{2}^{m}\sigma^{2}}{a_{2}^{2}a_{1}^{2}P_{S}\Omega_{1}\left(1-\beta \right)}\right)-Ei\left(\frac{-d_{2}^{m}\sigma^{2}}{a_{1}^{2}P_{S}\Omega_{1}\left(1-\beta \right)}\right)\right]\frac{e^{-\;\frac{d_{2}^{m}\sigma^{2}}{a_{1}^{2}P_{S}\Omega_{1}}}}{2\ln2} \end{array}$$

**Proof.**  See in Appendix A. □

### 3.3. Non-Energy Harvesting (NEH) as a Benchmark

We first denote $\rho =\frac{P_{S}}{\sigma_{}^{2}}$ as SNR at transmitter. Similarly with energy harvesting case, the outage probability of *D*1 in NEH case is given by:

(27) $$\begin{array}{cc} OP_{D1}^{NEH} & =\mathrm{Pr}\left(SNR_{D1,x1}^{NEH}<\gamma_{1}\right)\\ & =\mathrm{Pr}\left(\frac{a_{1}^{2}\rho^{2}{\left|h_{S}\right|}^{2}{\left|h_{D1}\right|}^{2}}{\rho {\left|h_{S}\right|}^{2}+\rho {\left|h_{D1}\right|}^{2}+1}<\gamma_{1}\right)\\ & =1-\exp\left(-\frac{2\gamma_{1}}{\Omega_{1}\rho a_{1}^{2}}\right)2\sqrt{\frac{\gamma_{1}}{\Omega_{1}\Omega_{2}\rho^{2}a_{1}^{2}}\left(\frac{\gamma_{1}}{a_{1}^{2}}+1\right)}K_{1}\left(2\sqrt{\frac{\gamma_{1}}{\Omega_{1}\Omega_{2}\rho^{2}a_{1}^{2}}\left(\frac{\gamma_{1}}{a_{1}^{2}}+1\right)}\right). \end{array}$$

Then the outage probability of *D*2 in NEH case is expressed by:

(28) $$\begin{array}{cc} OP_{D2}^{NEH}= & \mathrm{Pr}\left(SNR_{D2,x1}^{NEH}<\gamma_{2}\right)\mathrm{Pr}\left(SNR_{D2,x2}^{NEH}<\gamma_{2}\right)=J4\times J5 \end{array}$$

In which, it can be shown that:

(29) $$\begin{array}{cc} J4 & =\mathrm{Pr}\left(\frac{a_{1}^{2}\rho^{2}{\left|h_{S}\right|}^{2}{\left|h_{D1}\right|}^{2}}{a_{2}^{2}\rho^{2}{\left|h_{S}\right|}^{2}{\left|h_{D1}\right|}^{2}+\rho {\left|h_{S}\right|}^{2}+\rho {\left|h_{D1}\right|}^{2}+1}<\gamma_{2}\right)\\ & =1-e^{-\;\frac{2\gamma_{2}}{\Omega_{1}\rho \left(a_{2}^{2}-a_{1}^{2}\gamma_{2}\right)}}2\sqrt{\frac{\gamma_{2}\left(\gamma_{2}+\left(a_{2}^{2}-a_{1}^{2}\gamma_{2}\right)\right)}{\Omega_{1}\Omega_{2}\rho^{2}{\left(a_{2}^{2}-a_{1}^{2}\gamma_{2}\right)}^{2}}}K_{1}\left(2\sqrt{\frac{\gamma_{2}\left(\gamma_{2}+\left(a_{2}^{2}-a_{1}^{2}\gamma_{2}\right)\right)}{\Omega_{1}\Omega_{2}\rho^{2}{\left(a_{2}^{2}-a_{1}^{2}\gamma_{2}\right)}^{2}}}\right) \end{array}$$

(30) $$\begin{array}{cc} J5 & =\mathrm{Pr}\left(\frac{a_{2}^{2}\rho^{2}{\left|h_{S}\right|}^{2}{\left|h_{D1}\right|}^{2}}{\rho {\left|h_{S}\right|}^{2}+\rho {\left|h_{D1}\right|}^{2}+1}<\gamma_{2}\right)\\ & =1-\exp\left(-\frac{2\gamma_{2}}{\Omega_{1}\rho a_{2}^{2}}\right)2\sqrt{\frac{\gamma_{2}}{\Omega_{1}\Omega_{2}\rho^{2}a_{2}^{2}}\left(\frac{\gamma_{2}}{a_{2}^{2}}+1\right)}K_{1}\left(2\sqrt{\frac{\gamma_{2}}{\Omega_{1}\Omega_{2}\rho^{2}a_{2}^{2}}\left(\frac{\gamma_{2}}{a_{2}^{2}}+1\right)}\right) \end{array}$$

**Remark** **2.** *In this NEH case, the relay obtains higher power compared wireless power as EH case, then outage performance is expected to improve. It is hard to confirm it in such formula, but we can check via simulation as presentation in next section.*

## 4. Simulation Results

Unless otherwise stated, regarding on energy harvesting protocol, i.e., energy harvesting efficiency is set by η=1, source transmission power, $P_{S}=1$ (Joules/sec), and path loss exponent m=3 (which corresponds to an urban cellular network environment ). To perform the above outage events, separated probability components must be calculated. For simplicity, we assume that $\gamma_{1}=\gamma_{2}=\gamma =2^{2R}-1$ at the fixed rate *R*. In practice, different rates are assigned for different users, but in this study we set the source transmission rate, *R* = 1 (bits/sec/Hz) in the delay limited transmission mode for simple analysis. The distances *d*, $d_{1}$ and $d_{2}$ are normalized to unit value. For simplicity, similar noise variances at the relay and the destination nodes are assumed, i.e., different kinds of noise variance is set as $\sigma^{2}=0.01$ . Power allocation factors for NOMA $a_{1}^{2}=0.8,\;a_{2}^{2}=0.2$ except to specific simulation results. The mean values, $\Omega_{1},\Omega_{2}$ of the exponential random variables ${\left|h_{S}\right|}^{2},{\left|h_{D}\right|}^{2}$, respectively, are set to 1.

**Figure 2:**
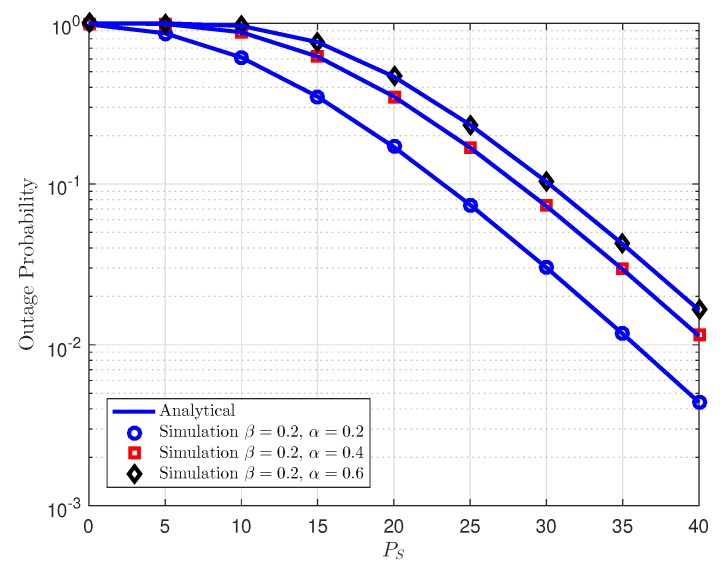
Outage probability vs. transmit power $P_{S}$ at *D*1 as varying power splitting fraction in TPSR.

**Figure 3:**
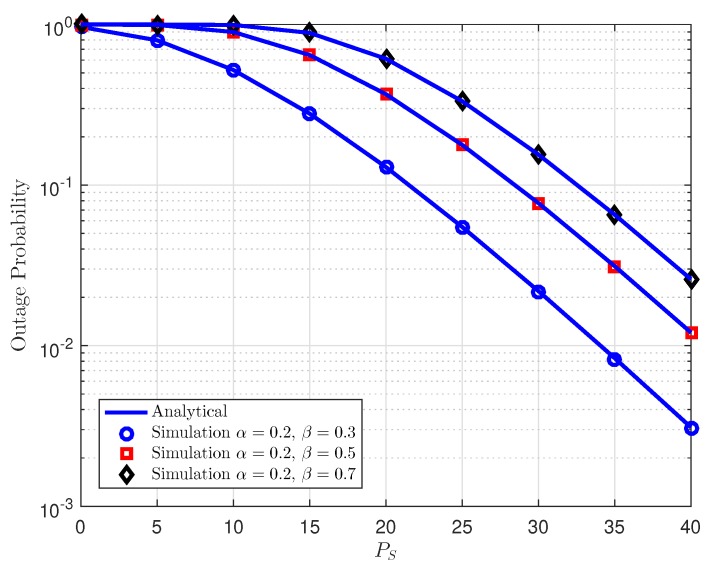
Outage probability vs. transmit power $P_{S}$ at *D*1 as varying power splitting fraction in time power switching-based relaying (TPSR).

Figure 2 plots the outage probability for cooperative NOMA with different power allocation factors for AF relaying, where energy harvesting fractions contribute to change outage performance shown as different curves. Observing the Figure 2, one can conclude that compared among three cases of EH-NOMA with AF relaying, the proposed scheme with higher time switching fraction allocation for energy harvesting can realize better outage performance as fixed power splitting factor is used. Furthermore, Figure 2 manifests that EH-NOMA can remarkably enhance the outage performance at high transmit power at source $P_{S}$. More importantly, the analytical curves match very well with Monte-Carlo results. While Figure 3 illustrates outage performance at *D*1 as varying power splitting factor. As can be seen clearly, increasing power splitting factor leads to better outage performance in case of fixed time switching fraction.

**Figure 4:**
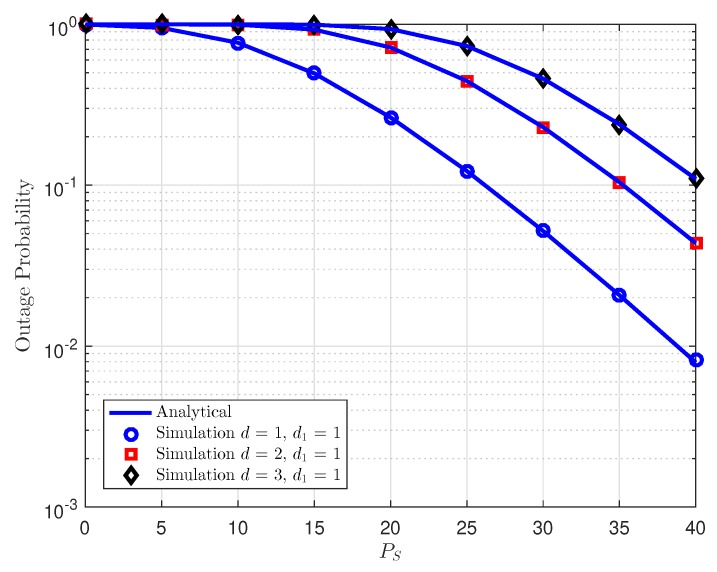
Outage probability vs. transmit power $P_{S}$ at *D*1 as distance between source and relay varies.

In Figure 4, by changing the relay location, it can be seen the outage performance will be affected by such parameter. Note that two users have different optimal locations. Since the source in NOMA assigns transmit power to the users, the ideal relay location for the user can impact system performance. In this case, as the relay locates far from the source the performance gets worse.

**Figure 5:**
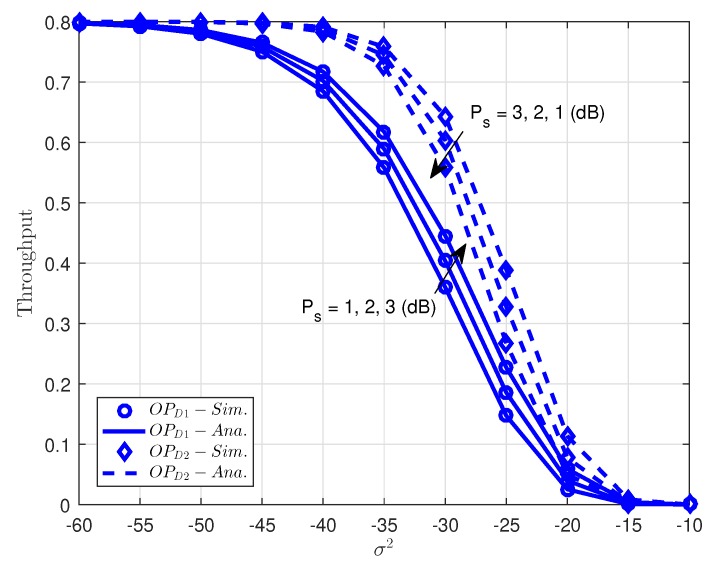
Comparison of outage performance at *D*1 and *D*2.

As can be seen in Figure 5, it shows the optimal throughput versus changing variance noise term in EH-NOMA in case of changing transmit power at source. It is noted that throughput can be shown as $\left(1-OP_{Di}\right)R\left(1-\alpha \right),i=1,2$ corresponding fixed rate *R*. As can be seen clearly, the proposed scheme with higher transmit power ($P_{S}=3\;\left(J/s\right)$ can realize better throughput performance due to more energy for signal processing. It is noted that noise term contributes to lowest throughput, especially at high noise −20 to −10 dB. In such a scenario, the outage probability of *D*2 outperforms the outage probability at *D*1. The reason is that more power allocated for *D*2 as principle of NOMA as concern before.

**Figure 6:**
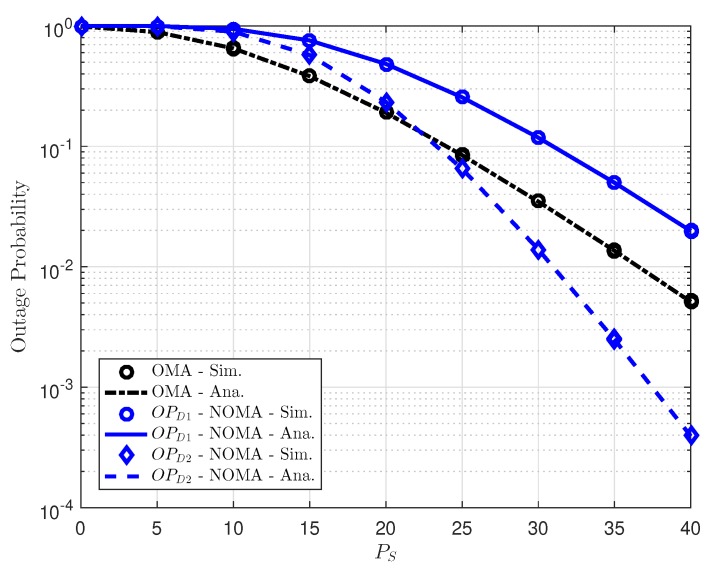
Comparison of outage performance at *D*1 and *D*2.

In Figure 6, we compare the outage performance for EH-NOMA with OMA scheme by using different users’ target rates and different numbers of relays. It can be observed from such experiment that OMA relaying schemes can achieve better outage performance than user *D*1 in NOMA scheme, but it is worse than outage performance of *D*2. Furthermore, we can also see that the outage performance in *D*2 of NOMA is significantly enhanced with high transmit power at source. In addition, Figure 6 also demonstrates that energy harvesting remains continuous operation of relay where has signal processing and outage performance at acceptable as reasonable selection of related parameters as in simulation result.

**Figure 7:**
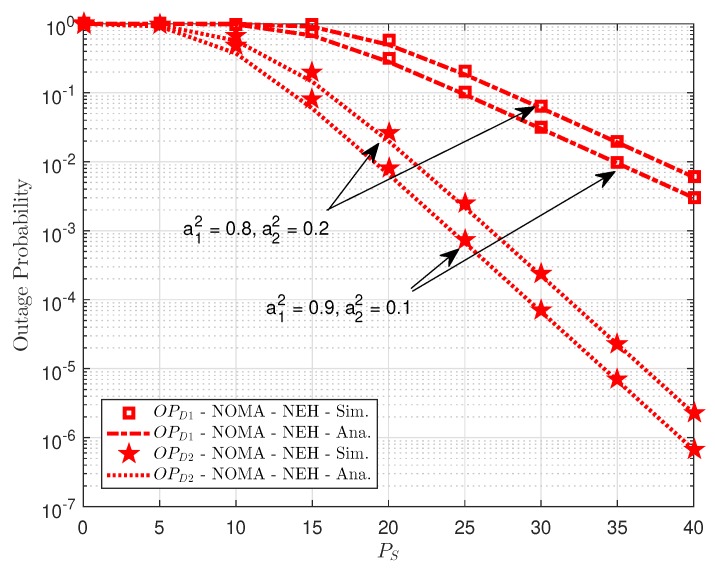
Comparison of Non-EH outage performance at *D*1 and *D*2.

**Figure 8:**
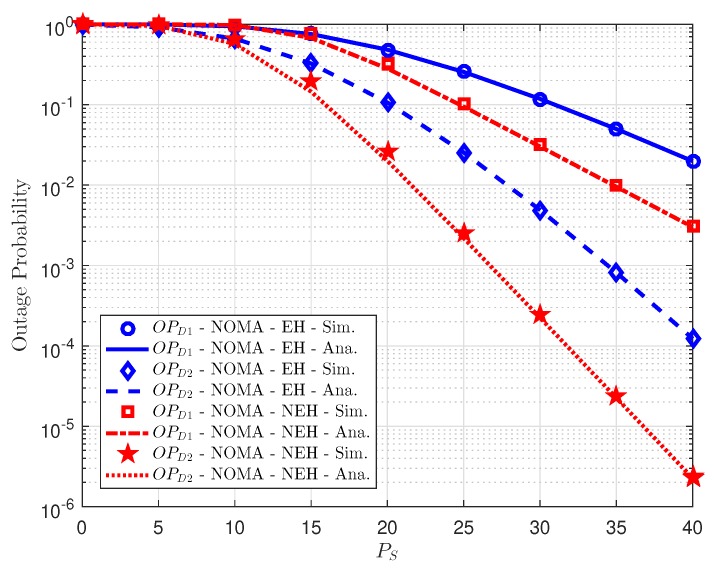
Comparison of Non-EH and EH outage performance at *D*1 and *D*2.

To evaluate system performance with NEH case, we illustrate outage performance on dedicated NEH situation as varying power allocation as in Figure 7. It can be seen that more power allocated to user *D*2 and higher outage performance can be achieved in the case that relay furnishes individual power (without energy harvesting). It is more important to perform such comparison in Figure 8, it compares EH case and NEH case, where NEH can be outperform than EH case due to higher amount power at relay used to transmit signal to *D*1 and *D*2. It is a consistent result with several experiments reported in [3,4,5,6].

**Figure 9:**
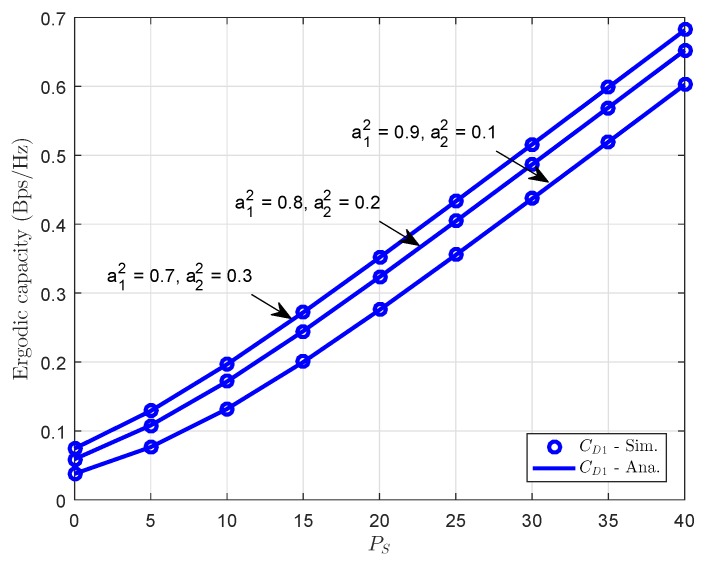
Ergodic capacity at *D*1 vs. transmit power $P_{S}$ with different power allocation factors.

**Figure 10:**
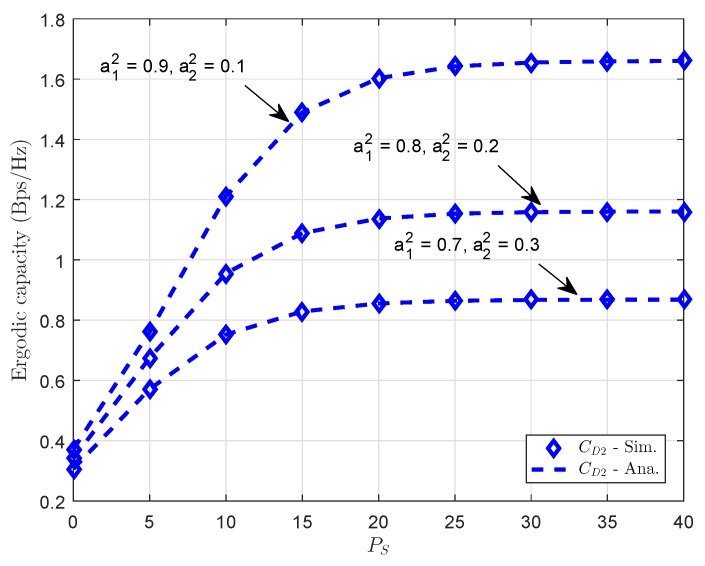
Ergodic capacity at *D*2 vs. transmit power $P_{S}$ with different power allocation factors.

Figure 9 and Figure 10 present the ergodic capacity versus transmit power at source with different power allocation factors in NOMA for each user. One can observe that *D*1 obtains the highest throughput since it has the higher power assigned among two users. We continue to study the ergodic performance versus the transmit SNR in Figure 8 and the power allocation factors are changed to evaluate its impacts. It can be seen clearly that increasing the transmit power can improve the ergodic capacity (throughput). The figure also demonstrates the existence of the throughput ceilings in the high transmit power $P_{S}$ region. This is due to the fact that the outage probability is approaching zero and the ergodic capacity is determined only by the targeted data rate. It can be confirmed that the analytic result that is obtained as previous section is valid.

**Figure 11:**
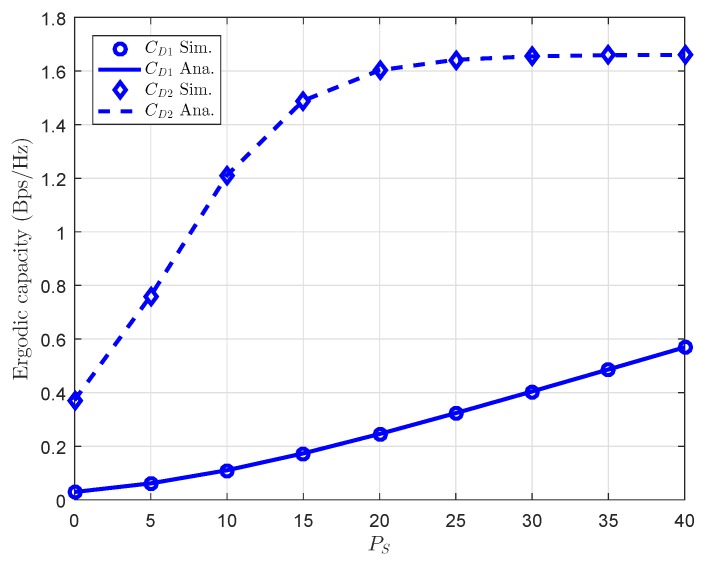
Comparison of ergodic capacity for *D*1 and *D*2.

Finally, performance gap in ergodic capacity of both *D*1 and *D*2 can be seen clearly in Figure 11. The main reason is that power allocation factor for each user is careful selected to guarantee quality of the proposed system.

## 5. Conclusions

In this paper, we have proposed AF relay schemes for EH-NOMA. For EH-NOMA relaying schemes, we have derived asymptotic analytical expressions for outage probability and ergodic capacity. The proposed cooperative NOMA schemes not only achieve reasonable performance but also yield better outage performance than OMA at specific scenarios. In addition, compared to different power allocation fractions, the proposed NOMA relay schemes can further improve the outage probability compared to OMA. It is confirmed that careful selection of energy harvesting protocol has important impacts on ergodic performance.

## Appendix A

**Proof** **of** **Lemma** **1.** It is required that $\psi {\left|h_{S}\right|}^{2}-\omega \neq 0.$ Therefore, $P_{out}^{TPSR}$ can be given in two cases below: In case of hS2<ωωψψ then:

(A1) $$P_{out}^{TPSR}=Pr\left({\left|h_{D}\right|}^{2}<\frac{\theta }{\psi {\left|h_{S}\right|}^{2}-\omega }\right).$$

In case of hS2>ωωψψ then:

(A2) $$P_{out}^{TPSR}=Pr\left({\left|h_{D}\right|}^{2}>\frac{\theta }{\psi {\left|h_{S}\right|}^{2}-\omega }\right)=1$$

Therefore, $P_{out}^{TPSR}$ can be formulated as:

(A3) $$\begin{array}{cc} P_{out}^{TPSR} & =\int_{0}^{\omega /\psi }Pr\left({\left|h_{D}\right|}^{2}>\frac{\theta }{\psi \gamma -\omega }\right)f_{{\left|h_{S}\right|}^{2}}\left(\gamma \right)d\gamma \;\\ & +\int_{\omega /\psi }^{\infty }Pr\left({\left|h_{D}\right|}^{2}<\frac{\theta }{\psi \gamma -\omega }\right)f_{{\left|h_{S}\right|}^{2}}\left(\gamma \right)d\gamma \;\;\;\; \end{array}$$

It is equivalent with following equation:

(A4) $$\begin{array}{c} P_{out}^{TPSR}=\int_{0}^{\omega /\psi }f_{{\left|h_{S}\right|}^{2}}\left(\gamma \right)d\gamma +\int_{\omega /\psi }^{\infty }\left(1-\exp\left\{-\frac{\theta }{\left(\psi x-\omega \right)\Omega_{2}}\right\}\right)f_{{\left|h_{S}\right|}^{2}}\left(\gamma \right)d\gamma \end{array}$$

where $\Omega_{1}$ and $\Omega_{2}$ are respectively average gain of channels ${\left|h_{S}\right|}^{2}$ and ${\left|h_{D}\right|}^{2},$fhS2γ=1Ω1e−γγΩ1Ω1 is (PDF) of random variables FhD2(γ)=PrhD2<γ=1−e−γγΩ2Ω2 stands for (CDF) of random variable ${\left|h_{D}\right|}^{2}.$ As a result, $P_{out}^{TPSR}$ can be expressed as:

(A5) $$P_{out}^{TPSR}=1-\frac{1}{\Omega_{1}}\int_{\omega /\psi }^{\infty }\exp\left\{-\frac{\gamma }{\Omega_{1}}-\frac{\theta }{\left(\psi \gamma -\omega \right)\Omega_{2}}\right\}d\gamma$$

We change to new variable μ=ψγ−ω. As a result, outage probability can be computed completely. This completes the proof. □

**Proof** **of** **Proposition** **4.** Due to such expression is resulted from following approximate SNR as below Interesting, noise term is very small as compared with term contains channel gain. This observation motivate us to produce the following approximation:

(A6) $$SNR_{D2,x1}\approx \frac{a_{1}^{2}}{a_{2}^{2}}$$

and:

(A7) $$SNR_{D2,x2}\approx \frac{a_{2}^{2}P_{S}{\left|h_{S}\right|}^{2}\left(1-\beta \right)}{d_{2}^{m}\sigma_{nR}^{2}}$$

It can be shown that ergodic capacity at *D*2 can be expressed by:

(A8) $$C_{D2}=\frac{1}{2\ln2}\int_{0}^{\infty }\frac{1-F_{V}\left(v\right)}{1+v}dv$$

Interestingly, we have following result:

(A9) $$F_{V}\left(y\right)=1-e^{-\frac{d_{2}^{m}\sigma_{nR}^{2}v}{a_{1}^{2}P_{S}\Omega_{1}}}U\left(\frac{a_{1}^{2}}{a_{2}^{2}}-v\right)$$

in which $U\left(\frac{a_{1}^{2}}{a_{2}^{2}}-y\right)$ denoted as step function. It equals to 1 if $a_{1}^{2}/a_{2}^{2}>v$ Based on ([27], Equation (3.352.1)) and applying some polynomial expansion manipulations:

(A10) $$\begin{array}{c} C_{D2}=\left[Ei\left(\frac{-d_{2}^{m}{\sigma_{nR}}^{2}}{a_{2}^{2}a_{1}^{2}P_{S}\Omega_{1}\left(1-\beta \right)}\right)-Ei\left(\frac{-d_{2}^{m}{\sigma_{nR}}^{2}}{a_{1}^{2}P_{S}\Omega_{1}\left(1-\beta \right)}\right)\right]\frac{1}{2\ln2}e^{-\frac{d_{2}^{m}{\sigma_{nR}}^{2}}{a_{1}^{2}P_{S}\Omega_{1}}} \end{array}$$

Finally, a high SNR approximation of the ergodic rate for *D*2 is written as in Proposition 4. It completes the proof. □
